# Multi-Organ Nutrigenomic Effects of Dietary Grapes in a Mouse Model

**DOI:** 10.3390/antiox12101821

**Published:** 2023-10-01

**Authors:** Asim Dave, Eun-Jung Park, John M. Pezzuto

**Affiliations:** 1 Division of Pharmaceutical Sciences, Arnold & Marie Schwartz College of Pharmacy and Health Sciences, Long Island University, Brooklyn, NY 11201, USA; davea3@mskcc.org (A.D.); eunjung.park@liu.edu (E.-J.P.); 2 Immunology Program, Memorial Sloan Kettering Cancer Center, New York, NY 10065, USA; 3 Department of Pharmaceutical and Administrative Science, College of Pharmacy and Health Sciences, Western New England University, Springfield, MA 01119, USA; 4 College of Pharmacy and Health Sciences, Western New England University, Springfield, MA 01119, USA; 5 Department of Medicine, UMass Chan Medical School—Baystate, Springfield, MA 01199, USA

**Keywords:** differentially expressed genes, liver, colon, kidney, ovary, pathway analysis, dietary influence on phenotype

## Abstract

As a whole food, the potential health benefits of table grapes have been widely studied. Some individual constituents have garnered great attention, particularly resveratrol, but normal quantities in the diet are meniscal. On the other hand, the grape contains hundreds of compounds, many of which have antioxidant potential. Nonetheless, the achievement of serum or tissue concentrations of grape antioxidants sufficient to mediate a direct quenching effect is not likely, which supports the idea of biological responses being mediated by an indirect catalytic-type response. We demonstrate herein with Hsd:ICR (CD-1^®^ Outbred, 18–24 g, 3–4 weeks old, female) mice that supplementation of a semi-synthetic diet with a grape surrogate, equivalent to the human consumption of 2.5 servings per day for 12 months, modulates gene expression in the liver, kidney, colon, and ovary. As might be expected when sampling changes in a pool of over 35,000 genes, there are numerous functional implications. Analysis of some specific differentially expressed genes suggests the potential of grape consumption to bolster metabolic detoxification and regulation of reactive oxygen species in the liver, cellular metabolism, and anti-inflammatory activity in the ovary and kidney. In the colon, the data suggest anti-inflammatory activity, suppression of mitochondrial dysfunction, and maintaining homeostasis. Pathway analysis reveals a combination of up- and down-regulation in the target tissues, primarily up-regulated in the kidney and down-regulated in the ovary. More broadly, based on these data, it seems logical to conclude that grape consumption leads to modulation of gene expression throughout the body, the consequence of which may help to explain the broad array of activities demonstrated in diverse tissues such as the brain, heart, eye, bladder, and colon. In addition, this work further supports the profound impact of nutrigenomics on mammalian phenotypic expression.

## 1. Introduction

The influence of diet on human health has been recognized over the millennia. From the time of Hippocrates (ca. 460 BC–ca. 370 BC), it has been clear that food has been considered good medicine and the converse under some situations. The health benefits of whole fruits and vegetables and a myriad of phytochemicals derived therefrom are widely recognized based on epidemiological investigations and clinical trials. In this context, we have been especially interested in the potential of table grapes to influence health and the mode of action.

Although some individual chemical constituents of grapes, particularly resveratrol [1], have been heavily studied, the grape is known to produce over 1600 phytochemicals [2]. Thus, regarding the potential of grapes to influence health, testing the product as a whole is more rational than testing individual components, especially when individual components are tested at concentrations unachievable through normal dietary means. Challenges of working with whole food, however, include the reproducibility of results experiment-by-experiment and year-by-year. This issue has been resolved by providing a surrogate whole-grape freeze-dried powder stored in vacuum-sealed packages. Showing good chemical stability when properly warehoused, the powder is composed of fresh seeded and seedless red, green, and black grapes that are grounded to retain their bioactive compounds. The standard product is subjected to chemical and microbial analyses to assure quality [3].

As a whole food, grapes have an impressive array of potential health benefits, including cardiovascular, atherosclerosis, inflammation, cancer, gastrointestinal, bone health, brain, joint, and vision [4]. Recently, regarding skin, we have reported that grape consumption can increase resistance to ultraviolet irradiation in human volunteers [5], with concomitant alterations in the metabolome [5] and the microbiome [6].

Mechanisms commonly associated with grapes and constituents of grapes have long been associated with antioxidant effects [7], interference with the NF-*κ*B signal transduction pathway [8], activation of Nrf2 signaling and induction of phase II detoxifying and antioxidant genes [9], etc. Reported responses include lowering LDL cholesterol oxidation and platelet aggregation, antiapoptotic, antimicrobial, antihypercholesterolemic, antiatherosclerotic, antiarrhythmic, and antidiabetic actions, among others [9]. Mechanistic underpinnings have been explored in many of these cases.

Considering the array of activities facilitated by the grape, it seems reasonable to speculate that, when taken as a whole, the plethora of chemical constitutes associated with this fruit may induce a broad-based, multifunctional response. Indeed, as demonstrated by Milella et al. [10], peripheral blood mononuclear cells from six human subjects who had consumed fresh table grapes identified 930 differentially expressed transcripts, some of which were associated with favorable processes such as immune response, DNA and protein repair, autophagy, and mitochondrial biogenesis. 

In our work with female C57BL6/J mice, as expected, the provision of a high-fat western-pattern diet led to significant increases in body weight and reduced lifespan. However, the addition of grapes to an isocaloric high-fat diet enhanced longevity and reduced fatty liver [11]. Simultaneously, significant alteration of hepatic gene expression was observed. Interestingly, the gene expression pattern of the mice receiving the high-fat diet containing grapes mapped closer to the standard diet containing grapes than the high-fat diet devoid of grapes. Further, with murine models, we have demonstrated that dietary grape consumption modulates gene expression in the brain, with corresponding behavior changes [12], as well as metabolomic alterations [13]. The alterations of gene expression induced by grape consumption likely provide a mechanistic underpinning.

In the current report, we examined the nutrigenomic effect of grapes more broadly. Similar to our work with grapes in the past [11,12,13], we elected to continue the studies with a murine model. In part, this was due to logistical considerations since, although mice consume a large amount of diet relative to their body weight, the rate of consumption is certainly less than rats, dogs, rabbits, or other larger mammals. We selected females, partly due to continuity with our past studies, as well as easing the burden of animal husbandry. Naturally, future work with male mice would be of value, but changing gender or evaluating both genders was not well-justified at the outset of this work. Finally, we chose to perform the study with CD-1 mice. Over the decades, CD-1 mice have been broadly used for research in toxicology, immunology, aging, and genetics. Here, however, we selected the CD-1 since it is an outbred strain. Although inbred mice have a more uniform genotype, which may allow for easier interpretation of results, this uniformity does not reflect the genetic diversity of the general human population. We considered this as an advantage of using CD-1 mice.

In our past work, using C57BL6/J mice, we primarily focused on the effect of grape consumption on the liver. In the present work, in addition to the liver, we assessed the effect of adding grapes to the diet on the gene expression in the colon, ovary, and kidney. Notable changes were observed in every case, indicating the global ability of grape consumption to alter the fundamental characteristics of mammalian cells and organs and suggesting a preeminent mechanism by which grape consumption may promote health.

## 2. Materials and Methods

### 2.1. Experimental Animals

Hsd:ICR (CD-1^®^ Outbred, 18–24 g, 3–4 weeks old, female) mice were obtained from Envigo RMS, LLC (Indianapolis, IN, USA). The mice were randomly divided into two groups of 10 mice each and were kept under a 12 h light–dark cycle. The dietary intervention study commenced immediately and continued for 12 months. Throughout the study, the animals had free access to the respective diet, except when fasting overnight in preparation for the next day’s sacrifice and organ harvesting. Water was available to the animals at all times. The animal protocol (16-01) was approved in advance by the Institutional Animal Care and Use Committee (IACUC) at Long Island University, Brookville, NY, USA. The body weight of the mice was monitored every two weeks. 

### 2.2. Diet

To assure the continuity and reproducibility of experimental and clinical studies performed with grapes, a surrogate powder provided in vacuum-sealed packets was obtained from the California Table Grape Commission (Fresno, CA, USA) and stored at −20 °C. The powder is composed of fresh seeded and seedless red, green, and black grapes that are freeze-dried and grounded to retain their bioactive compounds. The standard product is subjected to chemical and microbial analyses to assure quality [3].

A control diet (STD) and an isocaloric control diet supplemented with 5% (*w*/*w*) grape powder (STD5GP) (Table 1) were manufactured by Envigo (Madison, WI, USA). The formulations were based on the knowledge that the grape powder provides 3.71 kcal/g, with a composition of 3% fat, 88.6% carbohydrate (as a 1:1 mixture of fructose and glucose), 3.58% protein, and 9.73 g/kg of potassium. The diets were accordingly adjusted to ensure comparable kcal levels and stored at 4 °C.

### 2.3. Tissue Collection

The mice, at the age of 12 months, underwent euthanasia following an overnight fasting period. Euthanasia was performed using CO_2_. The liver, colon, kidneys, and ovaries were harvested and immediately submerged in an RNAlater™ stabilization solution (ThermoFisher Scientific, Waltham, CA, USA) for up to 12 h at room temperature. Subsequently, the samples were transferred to −20 °C until RNA extraction.

### 2.4. RNA Extraction and RNA Sequencing

Tissue samples for analysis were taken from the center of the colon, the distal part of the largest lobe of the liver, and the left lower part of the kidney. In addition, the entire left ovary was processed. Tissue homogenization was performed following the instructions of the manufacturer using 750 μL of QIAzol (Qiagen, Germantown, PA, USA) with an RNeasy 96 Universal Tissue kit (Qiagen, Germantown, PA, USA). To assess the quantity and quality of the extracted RNA samples, spectrophotometric measurements were taken using BioSpec-nano (Shimadzu, Tokyo, Japan), and the integrity of the RNA samples was evaluated via QIAxcel^®^ capillary electrophoresis. For the preparation of a “combined sample”, specimens derived from a respective tissue of each of the 10 mice in a group were pooled, ensuring that an equal amount of RNA from each mouse was added to the mixture. This yielded four samples from the STD group and four samples from the STD5GP group for analysis.

The library construction and RNA-seq were performed using Novogene (Sacramento, CA, USA). In brief, library concentration was first quantified using a Qubit 2.0 fluorometer (Life Technologies, Carlsbad, CA, USA) and then diluted to 1 ng/µL before checking insert size on an Agilent 2100 and quantifying to greater accuracy via quantitative PCR (Q-PCR) (library activity > 2 nM). Libraries are fed into HiSeq machines according to activity and expected data volume. The Novogene Corporation Inc. (Beijing, China) conducted paired-end (PE) 150 sequencing using the Illumina HiSeq platform, generating 20 million raw reads per sample. 

### 2.5. Pathway and GO Term Enrichment Analyses

Pathway analysis was performed for cancer gene pathways, Reactome pathways, PID pathway, and Biocarta using the Bioconductor package msigdb v.1.8.0.

### 2.6. Heat Map Generation

Heat maps were created using genes identified as differentially expressed with *q* < 0.05. Rows were centered and scaled using the *Z*-score. The hierarchical clusters were created using Ward’s linkage method.

### 2.7. Statistical Analyses

Statistical analyses were performed using two-tailed Student’s *t*-tests (Microsoft^®^ Excel, version 2206) unless otherwise indicated. A *p*-value of ≤0.05 was considered statistically significant. Differential expression analysis of two conditions/groups (two biological replicates per condition) was performed using the DESeq R package (1.18.0). DESeq provides statistical routines for determining differential expression in digital gene expression data using a model based on the negative binomial distribution. The resulting *p*-values were adjusted using the Benjamini and Hochberg approach for controlling the false discovery rate (fdr). Genes with *q* < 0.05 found via DESeq and Log2(Fold-change) of 1 were set as the thresholds for significant differential expression were assigned as differentially expressed.

## 3. Results and Discussion

### 3.1. Body Weight and Dose Selection

For the current study, we elected to supplement the murine diet with 5% standardized grape powder. For humans, a single serving of grapes is considered to be ¾ of one cup, which is approximately 124 g. Accordingly, based on body weight, daily consumption rates, and metabolic correction factors [14], it was estimated that supplementation of the mouse diet with 5% grape powder corresponds to the daily consumption of about 2.5 servings of fresh grapes by a human weighing 70 kg.

**Figure 1:**
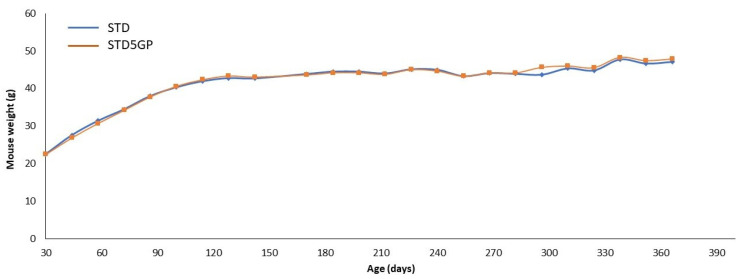
Average mouse body weight for groups consuming the STD and STD5GP. No significant differences were observed for the duration of the study.

In previous studies, we have demonstrated that the murine consumption rate of the STD and STD5GP does not differ [11]. This is clearly supported by the data shown in Figure 1. Body weight was determined every two weeks for the duration of the study. No significant difference was observed between the body weights of the two groups throughout. At the end of the study, the body weights of the STD and STD5GP groups were 41.46 ± 6.33 and 41.62 ± 6.60 g, respectively (*p* = 0.931, Student’s *t*-test). 

### 3.2. Differential Gene Expression 

To understand the multi-organ effect of grape consumption, we conducted RNA seq analysis from samples derived from the liver, colon, kidney, and ovary, as described in Section 2.4. Differential expression analysis was performed for each organ, with the criteria of *q* < 0.05 and Log2(Fold-change) > 1 for gene selection. A list of genes from the differentially expressed gene (DEG) analysis, showing genes both up- and down-regulated by consumption of the grape diet, is provided in Table S1.

When comparing the STD5GP vs. STD diet groups in the liver, we observed 27 DEGs (13 up-regulated and 14 down-regulated). The colon exhibited a higher number of DEGs, with 84 being differentially expressed (47 up-regulated; 37 down-regulated). In the kidney, we noted 21 DEGs (eight up-regulated and 13 down-regulated), and in the ovary, we found 49 DEGs (four up-regulated and 45 down-regulated). 

**Figure 2:**
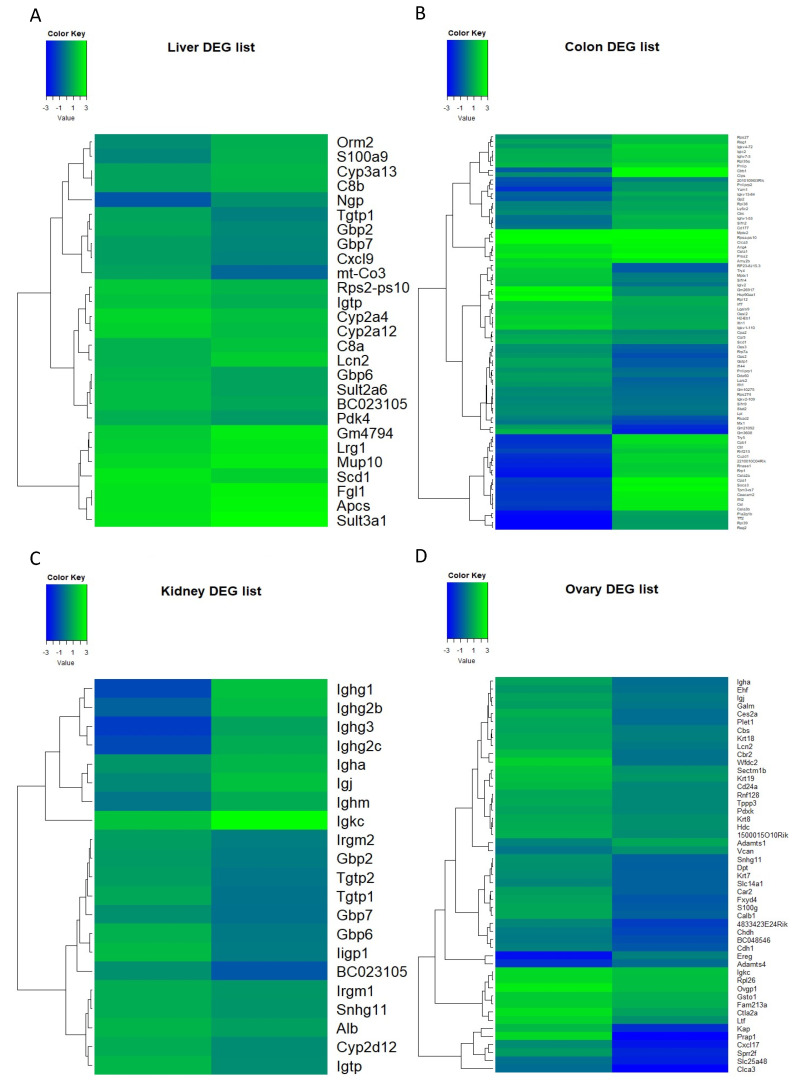
Heat maps illustrating differentially expressed genes (DEGs) with (**A**) liver, (**B**) colon, (**C**) kidney, and (**D**) ovary. Each heat map illustrates the gene expression levels with the *Z*-score, where blue indicates lower expression and green indicates higher expression. Rows are hierarchically clustered using Ward’s linkage method for pattern identification.

These data are exhibited as heat maps in Figure 2. The maps illustrate the shift in the gene expression across the tissues. Hierarchical clustering using Ward’s linkage was performed on the rows of the heat maps to minimize within-cluster variance for further analysis of the biological responses of genes. Figure 2A displays expression alterations within the liver, while Figure 2B displays changes occurring in the colon. Similarly, Figure 2C reveals the differential expression patterns in the kidney, and Figure 2D provides a visual representation of variations in the gene expression of the ovary. The genes featured in each heat map are drawn from tissue-specific DEG lists. This portrayal vividly captures the shift in expression patterns with the indicated tissues.

**Figure 3:**
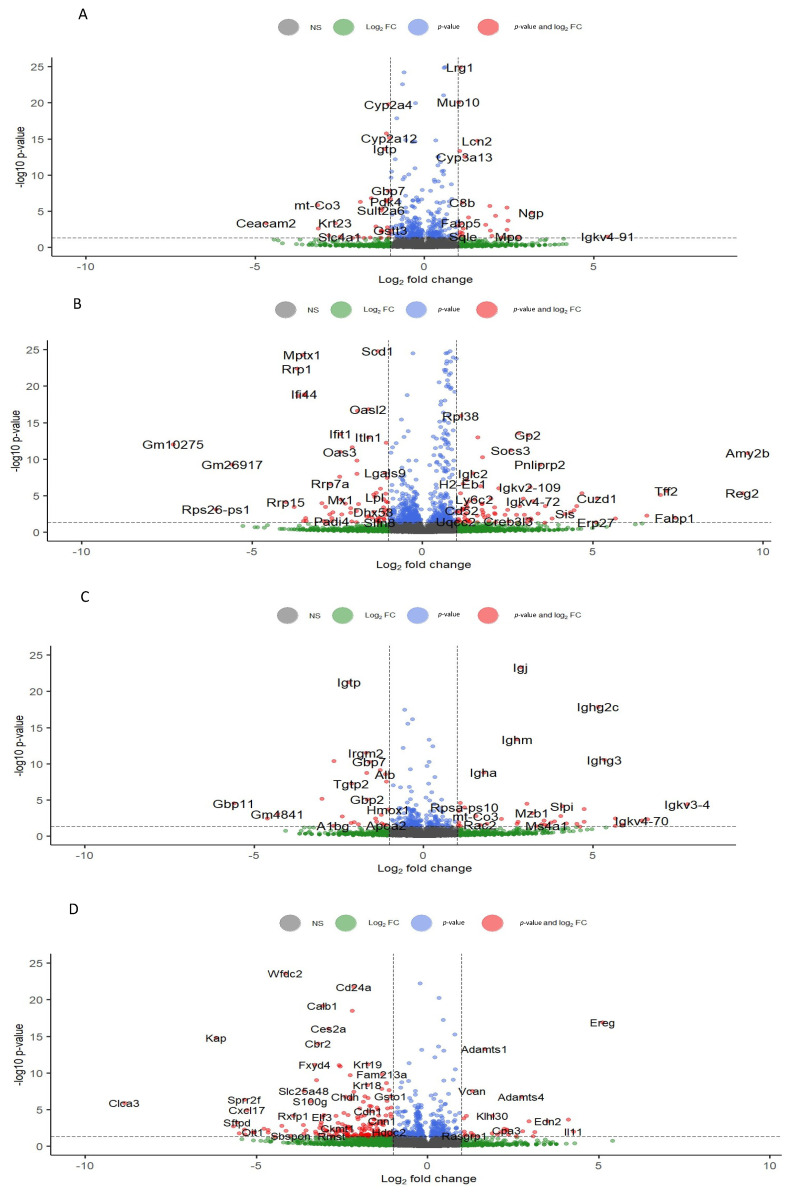
Volcano plots illustrating differentially expressed genes for (**A**) liver, (**B**) colon, (**C**) kidney, and (**D**) ovary. Significant changes were considered as those with a *p*-value < 0.05 and an absolute Log2(Fold-change) > 1 (delineated by the dotted lines).

We created volcano plots to visualize the entire gene list in our dataset and distinguish genes based on expression levels and statistical significance (Figure 3). The entire gene list is comprised of 35,275 genes, and the volcano plots show gene distribution for the liver (Figure 3A), colon (Figure 3B), kidney (Figure 3C), and ovary (Figure 3D). The plots further differentiate the genes based on their fold-change threshold Log2(Fold-change) > 1 and the significance value (*p* < 0.05). This allows us to identify genes with significant expression changes induced by the grape diet, separating them from genes with lower expression changes.

**Figure 4:**
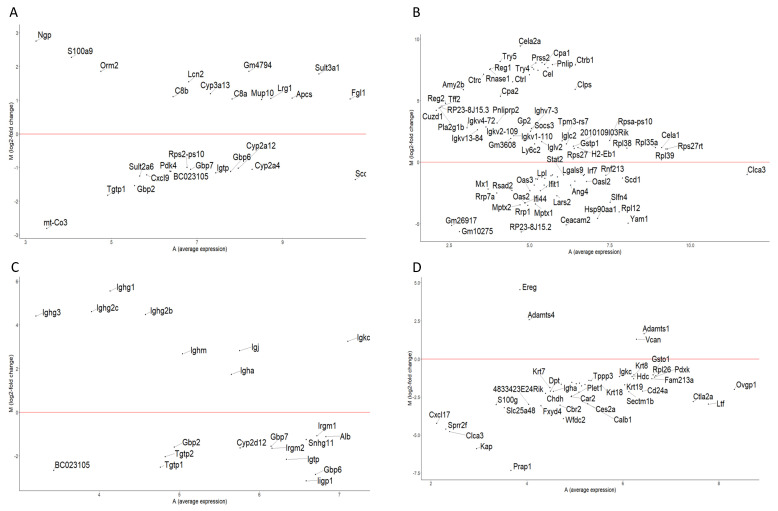
MA plots generated using the DEG list for (**A**) liver, (**B**) colon, (**C**) kidney, and (**D**) ovary.

Moreover, the DEG list from Table S1, which was visualized in the volcano plots (Figure 3), was used to generate MA plots (Figure 4). These plots illustrate the expression levels of the DEGs for each organ, namely, the liver (Figure 4A), colon (Figure 4B), kidney (Figure 4C), and ovary (Figure 4D). The distinct populations of genes on the MA plot represent the influence of grapes on the genes; some genes are clustered closely while others are uniquely positioned, suggesting varying magnitude of expression changes.

### 3.3. Pathway Analysis and Gene Enrichment

**Figure 5:**
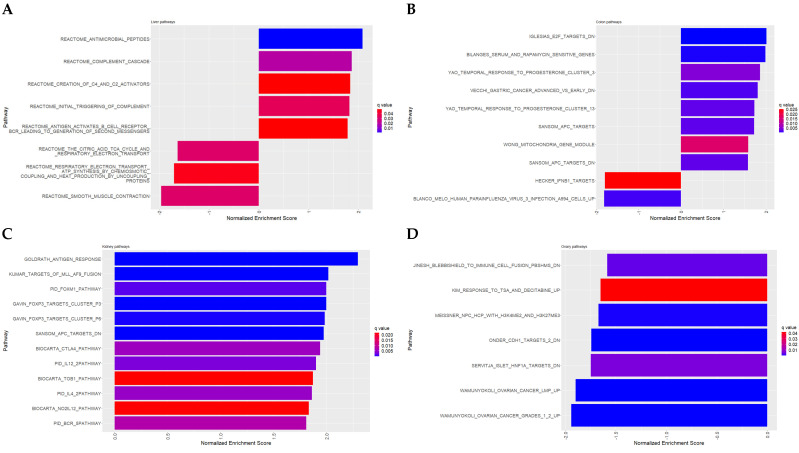
Pathway analysis showing significant enrichment (*q* < 0.05) in (**A**) liver, (**B**) colon, (**C**) kidney, and (**D**) ovary.

As an attempt to gain functional insight into the changes observed in various organs resulting from grape consumption, we conducted pathway analysis on the entire gene set. The pathways were analyzed for each subject organ: liver (Figure 5A), colon (Figure 5B), kidney (Figure 5C), and ovary (Figure 5D). Functional annotations of these pathways were derived from cancer gene pathways, Reactome pathways, PID pathways, and Biocarta. The data set used for the analysis was generated by comparing STD5GP vs. STD diet groups, and each specific gene set corresponding to a particular pathway was identified based on the ontology domain. The analysis provides the enrichment score and the statistical values, which were utilized to generate bar plots (Figure 5) for the significantly enriched pathways with *q* < 0.05. The pathways for each organ suggest the magnitude of enrichment influenced by grape consumption.

#### 3.3.1. Liver

- Pathway analysis

In the liver, pathways such as the citric acid cycle (TCA cycle) and the respiratory electron transport chain associated with ATP synthesis were found to be enriched in the STD group. With STD5GP, the triggering of complement and the complement cascade were enriched, suggesting enhanced capability of the liver to combat foreign invaders, including bacteria and viruses [15]. Further, the up-regulation of antimicrobial peptides and other immune activators suggests a homeostatic response to potential allergens within the tissue.

- Genes enriched

STD5GP exhibited up-regulated genes associated with various functions, including xenobiotic detoxification (*Sult3a1*) [16], protection through sulfation of benzene metabolites (*Gm4794*) [17], promotion of mitochondrial mitosis in response to liver injury (*FGL1*) [18], tumor suppressor gene (*APCs*) [19], and up-regulation of a component of the complement system (*C8a*) [20]. Down-regulation of the biosynthesis of monounsaturated fatty acids (*SCd1*) [21] was observed. 

#### 3.3.2. Colon

- Pathway analysis

In the colon, grape consumption induced the enrichment of various pathways. Specifically, the grape diet enhanced the enrichment of response to progesterone clusters 3 and 13, which are essential in regulating apoptosis. Additionally, the grape diet demonstrated enrichment of the mitochondria gene module, which is involved in energy production within the mitochondria [22]. Conversely, pathways associated with gastric cancer were down-regulated in response to the grape diet. The APC pathway exhibited a neutral response, while the E2F targets pathway showed down-regulation. Moreover, the grape diet resulted in the enrichment of the rapamycin-sensitive genes pathway.

- Genes enriched

STD5GP exhibited up-regulated genes associated with various functions, including CTRB1 [23] and clps [24], involved in the breakdown of food and the absorption of nutrients, and cpa1, which helps to protect the tissue from harmful bacteria and viruses through carboxypeptidase A1 [25]. Further, cel was up-regulated, which is responsible for regulating the pH of the colon [26], and Pnlip exhibited up-regulation, facilitating intestinal cholesterol absorption [27]. Furthermore, grape consumption was found to up-regulate the antiapoptotic gene Reg1, contributing to the regeneration of the gastric mucosal layer [28].

Conversely, the grape diet showed down-regulation of *ceacam2*, which is found in diseases such as ulcerative colitis and Crohn’s disease [29]. Further, down-regulation of *LARS2* was found in the STD5GP, which is shown to contribute to colorectal cancer [30]. Additionally, down-regulation was observed in *clca3*, which is associated with the prognosis of colon cancer [31].

#### 3.3.3. Kidney

- Pathway analysis

In the kidney, we observed enrichment of immune regulators, such as FOXP3 clusters, coupled with antigen response pathways. Further, we discovered enrichment of the IL-4 pathway, which plays a regulatory role in inflammation and fibrosis within the kidney [32]. The grape diet also demonstrated enrichment of the FOXM1 pathway, suggesting its pivotal involvement in repairing damaged kidney cells [33]. This enrichment coincided with the enrichment of the BCR-5 signaling pathway. Additionally, we observed enrichment of the IL-12 pathway, which is closely associated with Th-1 immune response [34]. This pathway may significantly combat infections caused by bacteria and other pathogens [35].

The consumption of grapes led to the enrichment of the Transducer of the ERBB2.1 (TOB1) pathway, which functions as a tumor suppressor and acts as a negative regulator of the ERBB2 receptor tyrosine kinase [36]. Additionally, the enrichment of the NO2IL12 pathway indicated the potential role of grapes in promoting host defense against intracellular microbial infections and controlling malignancy [37]. Furthermore, we observed enrichment of the CTLA4 pathway, highlighting the effect of grapes in preventing autoimmune diseases by inhibiting T cell activation [38].

- Genes enriched

In the kidney, we observed that grape consumption up-regulated genes associated with the production of the immunoglobulin kappa light chain (*IGKC*), along with the up-regulation of *IGJ*. This up-regulation enhances the ability of the kidney to combat infections [39,40]. Furthermore, grape consumption resulted in the up-regulation of *IgA*, which can potentiate a neutralizing effect on pathogens [41]. Additionally, the up-regulation of *Ighm* in the grape diet can further strengthen the immune defense of the kidney [42]. Notably, grape down-regulated *gbp2* and *snhg11* are associated with malignancy in renal cell carcinoma [43,44]. Further, down-regulation of *irgm1* and *irgm2* was observed, which can be associated with suppression of cell formation and Akt activation [45], further leading to suppressed carcinogenesis [45]. 

#### 3.3.4. Ovary

- Pathway analysis

In ovaries, we found significant enrichment of the ovarian cancer pathway, as well as the CDH1 targets and HNF1A pathways. Moreover, the enrichment of nuclear pore complex proteins and heat shock proteins in the STD diet provided a potential relationship between these proteins and ovarian cancer [46,47]. Furthermore, a down-regulation of the immune cell fusion PBShMS pathway was observed, suggesting its involvement in the susceptibility to autoimmune diseases [48].

- Genes enriched

In ovaries, we observed up-regulation of genes related to the process of ovulation, corpus luteum formation, and implantation of the embryo, as indicated by *adamts* [49] and *vcan* [50]. 

Conversely, there was a trend in down-regulation concerning genes related to ovarian cancer pathogenesis. Down-regulation of *ovgp1* [51], which is involved in the development of ovarian cancer, coupled with suppression of *CD24a* [52], which acts as a tumor-promoting factor by stimulating angiogenesis. Notably, *cxcl17* [53] was suppressed in the presence of STD5GP, suggesting reduced infiltration of antigen-presenting cells in the tissue. Additionally, our analysis revealed that the grape diet is a down-regulated promoter of methylation in ovarian cancer (*cdh1*) [54]. Moreover, TGF-beta signaling mediated ovarian cancer was attenuated by decreased expression of *igkc* [55]. Intriguingly, inhibition of genes such as *krt7* [56], *krt8* [56], *ehf* [57], *s100p* [58], *cbs* [59], *wfdc2* [60], *pdxk* [61], *fxyd* [62], *igj* [40], *snhg11* [63], and *calb1* [64] provides evidence of suppressive impact of the grape diet on progression of tumors in the ovary.

### 3.4. Potential Influence of Grape Consumption on Metabolic Pathways Associated with Health

As described above, the addition of grapes to the diet of mice influences gene expression in the liver, colon, kidney, and ovary, and presumably other organs as well. In related work, we have demonstrated physiological responses likely resulting from grape consumption related to the liver [11], brain [12], and longevity [11]. More broadly, dietary grapes have been demonstrated to mollify various disease states [4] in clinical trials and animal models.

**Figure 6:**
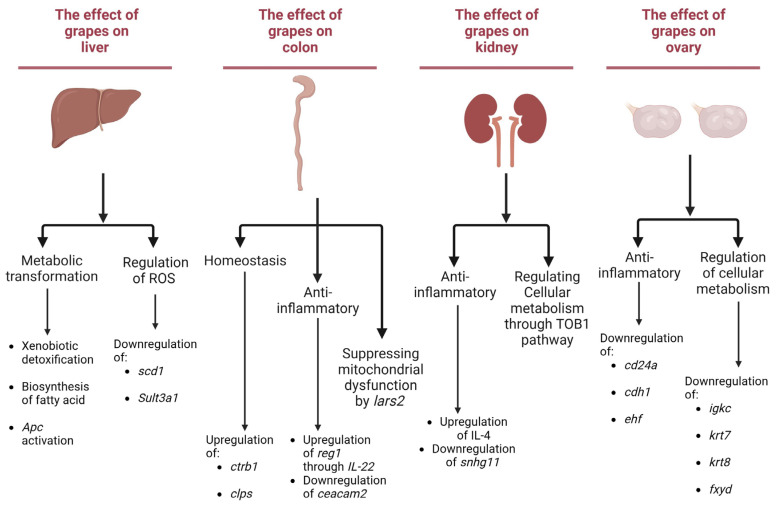
Graphical depiction of some key genes and pathways modulated by the grape diet in the liver, colon, kidney, and ovary.

In general, based on the alteration of gene expression modulated by grapes, it is logical to anticipate some cause-and-effect relationship. The main objective of this work was to examine the multi-organ nutrigenomic potential of grapes (Figure 6). Direct assessment of physiological responses was beyond the scope of the study. However, based on the accumulated data, it is possible to analyze the influence of grapes on metabolic pathways established as relevant to illness. Although additional investigations are required to prove such cause/effect relationships, some examples follow that provide suggestive evidence and a rationale for additional work.

#### 3.4.1. Liver-Associated Effects

In the liver, several facets of grape-related effects have emerged. First, grapes up-regulated the expression of *sult3a1*, which is associated with drug metabolism and pharmacokinetics (DMPK) responsible for xenobiotic detoxification [65]. This alteration in *sult3a1* expression is essential since it has been down-regulated in response to high-fat diets [65]. Importantly, the impact of *sult3a1* extends beyond metabolic processes, as its action in the liver contributes to reducing the risk of bladder cancer by decreasing the exposure of bladder tissue to toxins [66]. Furthermore, grapes exhibit an inhibitory effect on the biosynthesis of monounsaturated fatty acids via the enzyme *scd1* in the liver. This observation reinforces the potential of grapes in combatting adiposity [67]. Additionally, *scd1* plays a pivotal role in regulating ferroptosis [68], thereby reducing lipid reactive oxygen species [69]. Moreover, the ability of grapes to promote the activation of antigen-presenting cells (*APCs*) holds the potential for preventing hepatocellular carcinoma, particularly through the Wnt/β-catenin pathway [70]. This function is tied to the spatial organization within the portocentral axis, highlighting the significance of grapes in orchestrating metabolic and biotransformation processes within specific hepatic zones [19]. These multifaceted effects emphasize the interplay between metabolism and regulation of pathogenesis.

#### 3.4.2. Colon-Associated Effects

In the colon, grapes play a role in maintaining homeostasis through regulatory actions on *ctrb1* and *clps*. As discussed previously regarding involvement in food breakdown and nutrient absorption, it is established that *ctrb1* is a mediator of epithelial homeostasis, leading to amelioration of T cell-mediated colitis [71]. This protective effect is attributed to the nuclear receptor LRH-1, which binds to the proximal promoter of *ctrb1*, initiating downstream effects that promote cell survival and maintain epithelial homeostasis [71]. This function is further substantiated by the up-regulation of *reg1*, a downstream effector of IL-22 [72], and down-regulation of *ceacam2*, which is shown to be modified in inflammatory bowel disease (IBD) [73]. Further, grapes are involved in suppressing *lars2*, which has been linked with mitochondrial dysfunction [74]. This correlation is corroborated by our research findings that revealed an enrichment of down-regulated genes associated with the gastric cancer pathway, which suggests the impact of grapes on colon health, encompassing epithelial homeostasis and anti-inflammatory activity.

#### 3.4.3. Kidney-Associated Effects

In the kidney, the anti-inflammatory effects of grapes were indicated through the involvement of the IL-4 and FOXM1 pathways. In the context of acute kidney injury (AKI), it has been observed that IL-4 orchestrates the process of recovery, particularly within tubule-interstitial injury (TII) via albumin overload, without concurrent alterations in glomerular function [75]. Furthermore, the FOXM1 pathway is responsible for the proliferation of tubular epithelial cells following injury [76]. This occurs by regulating keratinocyte cell-cycle progression through the epidermal growth factor receptor (EGFR) [77]. Extending the scope of inquiry, we found down-regulation of genes such as *gbp2*, *snhg11*, *irgm1*, and *irgm2* with the grape diet. Specifically, *gbp2* has been observed to modulate the expression of programmed death-ligand 1 (PD-L1), along with the signal transducer and activator of transcription 1 [78]. Meanwhile, *snhg11* has been identified to play a role in DNA methylation [79] and an inducer of glycogen synthase kinase 3 beta (GSK-3β) ubiquitination, thereby contributing to the activation of the Wnt/β-catenin pathway [80]. Additionally, enrichment of the transforming growth factor beta-1 (TOB1) pathway provides evidence supporting the potential of the grape diet to remodel cell metabolism [81].

#### 3.4.4. Ovary-Associated Effects

In ovarian tissue, we observed enrichment of down-regulated genes associated with the ovarian cancer pathway. This down-regulation was primarily attributed to genes such as *ovgp1*, *CD24a*, *cdh1*, *igkc*, *krt7*, *krt8*, *ehf*, and *fxyd*. The down-regulation of *CD24a*, for instance, has been shown to inhibit the interaction with the inhibitory receptor known as sialic-acid-binding Ig-like lectin 10 (Siglec-10), which is expressed via tumor-associated macrophages. This suppression is significant in the context of cancer immunotherapy [82]. Furthermore, *cdh1* is known to be involved in DNA methylation [54], which was down-regulated by the grape diet. Additionally, genes *igkc*, *krt7*, and *krt8* play crucial roles in regulating the epithelial–mesenchymal transition (EMT), primarily through the TGF-β pathway [55,56], resulting in the regulating cellular metabolism [81]. Moreover, *ehf* has been found to reduce the phosphorylation of ERK and AKT, contributing to an anti-inflammatory effect. Simultaneously, the down-regulation of *fxyd5* illustrates a mechanism by which the regulation of cell metabolism is sustained, involving the regulation of TGF-β-driven EMT [83].

## 4. Conclusions

Using a well-defined semi-synthetic diet, we have previously shown the phytochemical components associated with the consumption of grapes as a whole food modulate gene expression in the liver [11] and brain [12]. We currently demonstrate that the modulatory effect is generalized throughout the body, as exemplified by investigation of the kidney, colon, and ovary, as well as liver (Figure 6). Some ramifications of these phenotypic changes are suggested through pathway analysis and consideration of the specific gene function. However, the implications are much broader. From a holistic point of view, the potential of dietary grapes to alter gene expression and corresponding downstream responses signifies a broad-based mechanism that may contribute to the pleiotropic activities mediated by grapes. Naturally, any influence of diet on genetic homeostasis depends on the diet as a whole. Nonetheless, the utilization of grapes in this capacity may be viewed as a prototype that exemplifies the power of dietary nutrigenomics.

## Figures and Tables

**Table 1:** Diet compositions.

		Control (STD)	Control + 5% Grape Powder (STD5GP)
		TD.160157	TD.160158
	Ingredient	(g/kg)	(g/kg)
1	Casein	195.0	192.94
2	DL-Methionine	3.0	3.0
3	Sucrose	191.0	191.0
4	Dextrose, anhydrous	66.45	44.3
5	Fructose	66.45	44.3
6	Corn starch	235.03	232.88
7	Maltodextrin	100.0	100.0
8	Anhydrous milkfat	30.0	29.85
9	Soybean oil	10.0	10.0
10	Cellulose	50.0	50.0
11	Mineral mix, AIN-76	35.0	35.0
12	Calcium carbonate	4.0	4.0
13	Potassium citrate, monohydrate	4.03	2.69
14	Vitamin mix, Teklad	10.0	10.0
15	Ethoxyquin, antioxidant	0.04	0.04
16	Grape powder, freeze-dried	0.0	50.0

## Data Availability

The datasets generated and analyzed for the current study are available in the National Center for Biotechnology Information (NCBI) repository. Bioproject accession number PRJNA1004855.

## Supplementary Materials

The following supporting information can be downloaded at https://www.mdpi.com/article/10.3390/antiox12101821/s1, Table S1: Genes present in the DEG list for different tissues.

## Abbreviations

AKI, acute kidney injury; APCs, antigen-presenting cells; DEG, differentially expressed gene; DMPK, drug metabolism and pharmacokinetics; EMT, epithelial–mesenchymal transition; GSK-3β, glycogen synthase kinase 3 beta; IACUC, institutional animal care and use committee; IGKC, immunoglobulin kappa light chain; IBD, inflammatory bowel disease; PE, paired-end; PD-L1, programmed death-ligand 1; siglec-10, sialic-acid-binding Ig-like lectin 10; STD, standard diet; STD5GP, standard diet containing 5% grape powder; EGFR, epidermal growth factor receptor; TOB1, transducer of ERBB2.1; TOB1, transforming growth factor beta-1; TII, tubule-interstitial injury (TII).
