# Supplementary material for: Multi-Organ Nutrigenomic Effects of Dietary Grapes in a Mouse Model
Source: Antioxidants (Basel). 2023 Oct 1;12(10):1821. doi: 10.3390/antiox12101821 (PMC10604885; doi:10.3390/antiox12101821)
Supplement: Supplementary file 1 [file antioxidants-12-01821-s001.zip › antioxidants-2647201-supplementary.pdf]

Multi-organ Nutrigenomic Effects of Dietary Grapes in a Mouse Model

Asim Dave [1,2], Eun-Jung Park [1,3] and John M. Pezzuto [4,5]*

[1] Division of Pharmaceutical Sciences, Arnold & Marie Schwartz College of Pharmacy and Health Sciences, Long Island University, Brooklyn, NY 11201, USA; davea3@mskcc.org; eunjung.park@liu.edu
[2] Current Address: Immunology Program, Memorial Sloan Kettering Cancer Center, New York, NY 10065, USA
[3] Current Address: Department of Pharmaceutical and Administrative Science, College of Pharmacy and Health Sciences, Western New England University, Springfield, MA 01119, USA
[4] College of Pharmacy and Health Sciences, Western New England University, Springfield, MA 01119, USA; john.pezzuto@wne.edu
[5] Department of Medicine, UMass Chan Medical School—Baystate, Springfield, MA 01199, USA
* Correspondence: john.pezzuto@wne.edu

Supplement 1. Data for the DEG list in liver, colon, kidney and ovary.

Table S1. Genes present in the DEG list for different tissues.
A. DEG list for liver.

Sr. No.	Gene Name	STD5GP vs STD	STD5GP vs STD Log2(Fold-change)	*q* value
1	*Apcs*	Up	1.06	$q < 10^{-4}$
2	*C8a*	Up	1.04	$q < 10^{-4}$
3	*Cyp3a13*	Up	1.2	$q < 10^{-4}$
4	*Fgl1*	Up	1.03	$q < 10^{-4}$
5	*Gm4794*	Up	1.86	$q < 10^{-4}$
6	*Lcn2*	Up	1.56	$q < 10^{-4}$
7	*Lrg1*	Up	1.05	$q < 10^{-4}$
8	*Mup10*	Up	1.02	$q < 10^{-4}$
9	*Sult3a1*	Up	1.78	$q < 10^{-4}$
10	*C8b*	Up	1.11	0.0001
11	*Orm2*	Up	1.93	0.0004
12	*S100a9*	Up	2.43	0.0006
13	*Ngp*	Up	3.16	0.0027
14	*Cyp2a12*	Down	-1.03	$q < 10^{-4}$
15	*Cyp2a4*	Down	-1.06	$q < 10^{-4}$
16	*Gbp2*	Down	-1.57	$q < 10^{-4}$
17	*Gbp6*	Down	-1.12	$q < 10^{-4}$
18	*Gbp7*	Down	-1.07	$q < 10^{-4}$
19	*Igtp*	Down	-1.17	$q < 10^{-4}$

20	*Scd1*	Down	-1.36	$q < 10^{-4}$
21	*BC023105*	Down	-1.13	0.0001
22	*Pdk4*	Down	-1.13	0.0001
23	*Rps2-ps10*	Down	-1.01	0.0001
24	*Tgtp1*	Down	-1.89	0.0001
25	*mt-Co3*	Down	-3.15	0.0003
26	*Cxcl9*	Down	-1.24	0.0008
27	*Sult2a6*	Down	-1.28	0.0014

B. DEG list for colon.

Sr. No.	Gene Name	STD5GP vs STD	STD5GP vs STD Log2(Fold-change)	*q* value
1	*2010109I03Rik*	Up	1.62	$q < 10^{-4}$
2	*2210010C04Rik*	Up	8.05	$q < 10^{-4}$
3	*Amy2b*	Up	9.54	$q < 10^{-4}$
4	*Cel*	Up	8.18	$q < 10^{-4}$
5	*Cela1*	Up	1.19	$q < 10^{-4}$
6	*Cela2a*	Up	9.45	$q < 10^{-4}$
7	*Cela3b*	Up	8.47	$q < 10^{-4}$
8	*Clps*	Up	6.03	$q < 10^{-4}$
9	*Cpa1*	Up	8.56	$q < 10^{-4}$
10	*Cpa2*	Up	6.02	$q < 10^{-4}$
11	*Cpb1*	Up	8.27	$q < 10^{-4}$
12	*Ctrb1*	Up	8.17	$q < 10^{-4}$
13	*Ctrc*	Up	8.48	$q < 10^{-4}$
14	*Ctrl*	Up	7.32	$q < 10^{-4}$
15	*Gp2*	Up	3.09	$q < 10^{-4}$
16	*Gstp1*	Up	1.3	$q < 10^{-4}$
17	*H2-Eb1*	Up	1.19	$q < 10^{-4}$
18	*Ighv7-3*	Up	2.83	$q < 10^{-4}$
19	*Iglc2*	Up	1.49	$q < 10^{-4}$
20	*Iglv2*	Up	1.7	$q < 10^{-4}$
21	*Pnlip*	Up	8.42	$q < 10^{-4}$
22	*Pnliprp1*	Up	7.73	$q < 10^{-4}$
23	*Pnliprp2*	Up	3.45	$q < 10^{-4}$
24	*Prss2*	Up	8.78	$q < 10^{-4}$
25	*Reg1*	Up	9.07	$q < 10^{-4}$
26	*Rnase1*	Up	8.03	$q < 10^{-4}$
27	*Rpl35a*	Up	1.18	$q < 10^{-4}$
28	*Rpl38*	Up	1.13	$q < 10^{-4}$
29	*Rpl39*	Up	1.05	$q < 10^{-4}$
30	*Rps27rt*	Up	1.07	$q < 10^{-4}$
31	*Rpsa-ps10*	Up	1.76	$q < 10^{-4}$
32	*Socs3*	Up	2.6	$q < 10^{-4}$

33	*Tpm3-rs7*	Up	1.76	$q < 10^{-4}$
34	*Try4*	Up	8.91	$q < 10^{-4}$
35	*Try5*	Up	8.33	$q < 10^{-4}$
36	*Igkv1-110*	Up	2.24	0.0001
37	*Igkv2-109*	Up	3.15	0.0001
38	*Tff2*	Up	7.17	0.0002
39	*Reg2*	Up	9.39	0.0004
40	*Rps27*	Up	1.11	0.0004
41	*RP23-8J15.3*	Up	4.67	0.0004
42	*Pla2g1b*	Up	6.99	0.0007
43	*Cuzd1*	Up	5.12	0.0018
44	*Gm3608*	Up	2	0.0019
45	*Igkv13-84*	Up	2.97	0.002
46	*Ly6c2*	Up	1.53	0.0025
47	*Igkv4-72*	Up	3.25	0.0043
48	*Ang4*	Down	-1.9	$q < 10^{-4}$
49	*Ceacam2*	Down	-5.23	$q < 10^{-4}$
50	*Clca3*	Down	-1.03	$q < 10^{-4}$
51	*Ddx60*	Down	-2.06	$q < 10^{-4}$
52	*Gm10275*	Down	-7.3	$q < 10^{-4}$
53	*Gm26917*	Down	-5.58	$q < 10^{-4}$
54	*Hsp90aa1*	Down	-4.61	$q < 10^{-4}$
55	*Ifi44*	Down	-3.46	$q < 10^{-4}$
56	*Ifit1*	Down	-2.39	$q < 10^{-4}$
57	*Ifit2*	Down	-1.93	$q < 10^{-4}$
58	*Ighv1-53*	Down	-1.92	$q < 10^{-4}$
59	*Irf7*	Down	-1.03	$q < 10^{-4}$
60	*Itln1*	Down	-1.58	$q < 10^{-4}$
61	*Lars2*	Down	-2.8	$q < 10^{-4}$
62	*Lgals9*	Down	-1.1	$q < 10^{-4}$
63	*Mptx1*	Down	-3.52	$q < 10^{-4}$
64	*Mptx2*	Down	-3.66	$q < 10^{-4}$
65	*Oas2*	Down	-3.46	$q < 10^{-4}$
66	*Oas3*	Down	-2.42	$q < 10^{-4}$
67	*Oasl2*	Down	-1.59	$q < 10^{-4}$
68	*Rnf213*	Down	-1.07	$q < 10^{-4}$
69	*RP23-8J15.2*	Down	-6.12	$q < 10^{-4}$
70	*Rpl12*	Down	-4.08	$q < 10^{-4}$
71	*Rrp1*	Down	-3.67	$q < 10^{-4}$
72	*Rrp7a*	Down	-2.71	$q < 10^{-4}$
73	*Rsad2*	Down	-2.43	$q < 10^{-4}$
74	*Scd1*	Down	-1.31	$q < 10^{-4}$
75	*Slfn4*	Down	-3.29	$q < 10^{-4}$
76	*Yam1*	Down	-4.99	$q < 10^{-4}$
77	*Car3*	Down	-1.23	0.0001

78	*Slfn9*	Down	-1.35	0.0004
79	*Stat2*	Down	-1.07	0.0005
80	*Cd177*	Down	-1.44	0.0006
81	*Lpl*	Down	-1.4	0.0013
82	*Slfn2*	Down	-1.13	0.0022
83	*Mx1*	Down	-2.39	0.0026
84	*Gm21092*	Down	-1.06	0.0047

C. DEG list for kidney.

Sr. No.	Gene Name	STD5GP vs STD	STD5GP vs STD Log2(Fold-change)	*q* value
1	*Igha*	Up	1.77	$q < 10^{-4}$
2	*Ighg1*	Up	6.24	$q < 10^{-4}$
3	*Ighg2b*	Up	4.77	$q < 10^{-4}$
4	*Ighg2c*	Up	5.16	$q < 10^{-4}$
5	*Ighg3*	Up	5.34	$q < 10^{-4}$
6	*Ighm*	Up	2.76	$q < 10^{-4}$
7	*Igj*	Up	2.87	$q < 10^{-4}$
8	*Igkc*	Up	3.27	$q < 10^{-4}$
9	*Alb*	Down	-1.13	$q < 10^{-4}$
10	*Cyp2d12*	Down	-1.67	$q < 10^{-4}$
11	*Gbp6*	Down	-2.88	$q < 10^{-4}$
12	*Gbp7*	Down	-1.59	$q < 10^{-4}$
13	*Igtp*	Down	-2.19	$q < 10^{-4}$
14	*Iigp1*	Down	-3.19	$q < 10^{-4}$
15	*Irgm1*	Down	-1.1	$q < 10^{-4}$
16	*Irgm2*	Down	-1.69	$q < 10^{-4}$
17	*Snhg11*	Down	-1.28	$q < 10^{-4}$
18	*Tgtp1*	Down	-2.64	$q < 10^{-4}$
19	*Tgtp2*	Down	-2.12	$q < 10^{-4}$
20	*BC023105*	Down	-2.99	0.0032
21	*Gbp2*	Down	-1.66	0.0036

D. DEG list for ovary.

Sr. No.	Gene Name	STD5GP vs STD	STD5GP vs STD Log2(Fold-change)	*q* value
1	*Adamts1*	Up	1.67	$q < 10^{-4}$
2	*Ereg*	Up	5.1	$q < 10^{-4}$
3	*Vcan*	Up	1.31	$q < 10^{-4}$
4	*Adamts4*	Up	2.75	0.0001
5	*4833423E24Rik*	Down	-3.24	$q < 10^{-4}$
6	*Calb1*	Down	-3.05	$q < 10^{-4}$
7	*Car2*	Down	-2.58	$q < 10^{-4}$
8	*Cbr2*	Down	-3.19	$q < 10^{-4}$

9	*Cd24a*	Down	-2.16	$q < 10^{-4}$
10	*Ces2a*	Down	-2.89	$q < 10^{-4}$
11	*Ctla2a*	Down	-2.82	$q < 10^{-4}$
12	*Fam213a*	Down	-1.32	$q < 10^{-4}$
13	*Fxyd4*	Down	-3.28	$q < 10^{-4}$
14	*Kap*	Down	-6.18	$q < 10^{-4}$
15	*Krt18*	Down	-1.76	$q < 10^{-4}$
16	*Krt19*	Down	-1.73	$q < 10^{-4}$
17	*Lcn2*	Down	-2.15	$q < 10^{-4}$
18	*Ltf*	Down	-3	$q < 10^{-4}$
19	*Ovgp1*	Down	-2.03	$q < 10^{-4}$
20	*Pdxk*	Down	-1.2	$q < 10^{-4}$
21	*Plet1*	Down	-2.55	$q < 10^{-4}$
22	*Prap1*	Down	-9.85	$q < 10^{-4}$
23	*Rnf128*	Down	-1.33	$q < 10^{-4}$
24	*Rpl26*	Down	-1.12	$q < 10^{-4}$
25	*Sectm1b*	Down	-2.2	$q < 10^{-4}$
26	*Slc25a48*	Down	-3.62	$q < 10^{-4}$
27	*Snhg11*	Down	-2.26	$q < 10^{-4}$
28	*Wfdc2*	Down	-4.15	$q < 10^{-4}$
29	*BC048546*	Down	-1.83	0.0001
30	*Cbs*	Down	-1.74	0.0001
31	*Chdh*	Down	-2.4	0.0001
32	*Dpt*	Down	-2.24	0.0001
33	*Gsto1*	Down	-1.07	0.0001
34	*Igha*	Down	-2.24	0.0001
35	*Sprr2f*	Down	-5.35	0.0001
36	*Hdc*	Down	-1.19	0.0002
37	*S100g*	Down	-3.43	0.0003
38	*Igj*	Down	-1.88	0.0003
39	*Clca3*	Down	-8.84	0.0004
40	*Ehf*	Down	-1.69	0.0009
41	*Galm*	Down	-1.64	0.0009
42	*Tppp3*	Down	-1.47	0.0014
43	*Krt8*	Down	-1.07	0.0014
44	*Krt7*	Down	-1.99	0.0017
45	*Igkc*	Down	-1.18	0.0017
46	*1500015O10Rik*	Down	-1.45	0.0025
47	*Cxcl17*	Down	-5.27	0.0027
48	*Cdh1*	Down	-1.74	0.0038
49	*Slc14a1*	Down	-1.61	0.0038
